# Spatial heterogeneity of oxygenation and haemodynamics in breast cancer resolved in vivo by conical multispectral optoacoustic mesoscopy

**DOI:** 10.1038/s41377-020-0295-y

**Published:** 2020-04-13

**Authors:** Jiao Li, Andrei Chekkoury, Jaya Prakash, Sarah Glasl, Paul Vetschera, Benno Koberstein-Schwarz, Ivan Olefir, Vipul Gujrati, Murad Omar, Vasilis Ntziachristos

**Affiliations:** 1 School of Precision Instruments and Optoelectronics Engineering, Tianjin University, No.92, Weijin Road, Nankai District, 300072 Tianjin, China; 2 Institute of Biological and Medical Imaging, Helmholtz Zentrum München, Ingolstädter Landstr. 1, D-85764 Neuherberg, Germany; 3 Chair of Biological Imaging, TranslaTUM, Technische Universität München, Ismaningerstr. 22, D-81675 Munich, Germany; 4 Department of Instrumentation and Applied Physics, Indian Institute of Science Bangalore, CV Raman Rd, Bengaluru, 560012 Karnataka India

**Keywords:** Photoacoustics, Biophotonics, Imaging and sensing

## Abstract

The characteristics of tumour development and metastasis relate not only to genomic heterogeneity but also to spatial heterogeneity, associated with variations in the intratumoural arrangement of cell populations, vascular morphology and oxygen and nutrient supply. While optical (photonic) microscopy is commonly employed to visualize the tumour microenvironment, it assesses only a few hundred cubic microns of tissue. Therefore, it is not suitable for investigating biological processes at the level of the entire tumour, which can be at least four orders of magnitude larger. In this study, we aimed to extend optical visualization and resolve spatial heterogeneity throughout the entire tumour volume. We developed an optoacoustic (photoacoustic) mesoscope adapted to solid tumour imaging and, in a pilot study, offer the first insights into cancer optical contrast heterogeneity in vivo at an unprecedented resolution of <50 μm throughout the entire tumour mass. Using spectral methods, we resolve unknown patterns of oxygenation, vasculature and perfusion in three types of breast cancer and showcase different levels of structural and functional organization. To our knowledge, these results are the most detailed insights of optical signatures reported throughout entire tumours in vivo, and they position optoacoustic mesoscopy as a unique investigational tool linking microscopic and macroscopic observations.

## Optoacoustic imaging: Revealing the details of tumour patterns

A technique that can image the entire tumour volume with high resolution may help oncologists optimize specific treatments for breast cancer. Jiao Li (Tianjin University, China), Vasilis Ntziachristos (Technical University of Munich, Germany), and colleagues have designed a multispectral optoacoustic mesoscope (MSOM) that illuminates millimetre-sized tumours with laser light of various wavelengths, and detects the ultrasound waves generated by internal absorbers, such as haemoglobin, or external nanoparticle probes. By reconstructing the ultrasound signals over multiple frequencies, the team produced 3D images of features that included the vascular network of a tumour with micrometre-scale detail. Experiments with live mice demonstrated that specific tumours could be identified through differences in spatial patterns, such as altered oxygen levels between tumour cores and peripheries. The study highlights the power of MSOM as a tool for preclinical cancer studies.

## Introduction

Tumours contain genetically and phenotypically distinct cell subpopulations exposed to heterogeneous vascularization and signalling microenvironments^1^. Understanding the tumour microenvironment and its heterogeneity are key to elucidating cancer progression, metastasis and response to treatment. In addition to genetic heterogeneity, spatial heterogeneity may play a vital role in tumour growth, metastasis or treatment, especially if different parts of a tumour respond in different ways^2^.

Intravital (in vivo) microscopy is commonly used in cancer research and has revealed critical information about cell motility that leads to metastasis, about the function of immune cells within tumours and their role in inhibiting or promoting tumour growth, and about the different responses of cell populations within tumours to the same drug^3–5^ or to radiation therapy^6^. Nevertheless, optical microscopy is limited by photon scattering^7^ and can assess small volumes of only a few hundred cubic microns^1,8^, so it is not suited for comprehensively studying the morphology, functionality and the overall heterogeneity of an entire tumour volume^9^. Surgical removal of tissue and the implantation of “optical windows”^10^ allow microscopic observations of deeper tumour areas but similarly limit the comprehensive visualization of entire tumours. The need to image larger volumes has led to the development of several alternatives. Three-dimensional light-sheet microscopy^11,12^ offers the possibility of volumetric imaging. However, it requires chemical processing of specimens to make them transparent and is therefore not suited for in vivo applications or imaging of tissue physiology.

As an alternative to optical methods, optoacoustic imaging has demonstrated high-resolution imaging of optical contrast through several millimetres to centimetres of tissue^13–16^. There are two major optoacoustic imaging techniques. Optical-resolution optoacoustic (photoacoustic) microscopy uses focused light to excite ultrasound responses, but its depth and resolution are similar to those of optical (photonic) microscopy since both modalities form images limited by optical diffraction. Conversely, acoustic-resolution optoacoustic imaging can be performed using unfocused illumination, in which case the depth and resolution depend strongly on the characteristics of the ultrasound detection arrangement employed and the ultrasound frequency band collected. The combination of deep-seated high-resolution imaging with multi-wavelength illumination gives rise to multispectral optoacoustic tomography (MSOT), which can resolve the spectral signatures of intrinsic chromophores and extrinsically administered absorbers. MSOT has been consequently employed to visualize and quantitate the oxygenation status of tumours^17–19^ and resolve angiogenesis and a number of external agents targeting physiological and molecular tumour parameters in animals and humans^19–25^. Nevertheless, current optoacoustic imaging systems do not allow high-resolution imaging of tumour heterogeneity. Tomographic systems using detector arrays have allowed three-dimensional imaging that yielded cross-sectional images throughout entire tumours^18^, but the resolutions achieved have been on the order of 100–500 μm. These resolutions prevent the detailed observation of tumour spatial heterogeneity. Systems that raster scan an ultrasound detector operating at higher frequencies than detector arrays allow higher resolution imaging but offer only limited-view images of superficial vasculature^20,21,23,24,26,27^, typically rendered as two-dimensional maximum intensity projections (MIPs).

Herein, we introduce multispectral optoacoustic mesoscopy (MSOM) with a novel system that combines three-dimensional performance with high-resolution imaging. We identified ~1–30 MHz as the optimal ultrasound detector bandwidth offering the optimal trade-off between resolution and cross-sectional imaging of entire tumours. We implemented this frequency band in a conical geometry enabling optical views of tumour heterogeneity with unprecedented detail. By applying MSOM at multiple wavelengths, we studied, for the first time, patterns of the spatial heterogeneity of tumour oxygenation and validated these patterns through co-registration with patterns of vascularization and permeability throughout the entire tumour mass. By applying the technique to three breast cancer models in mice, we studied intra- and inter-tumour heterogeneity and showed how in vivo MSOM imaging can be applied to quantitatively characterize the spatial heterogeneity in breast cancer based on functional tumour parameters in vivo. We discuss the unique performance achieved by our conical MSOM implementation and the implications for providing new possibilities in cancer research.

## Results

**Fig. 1:**
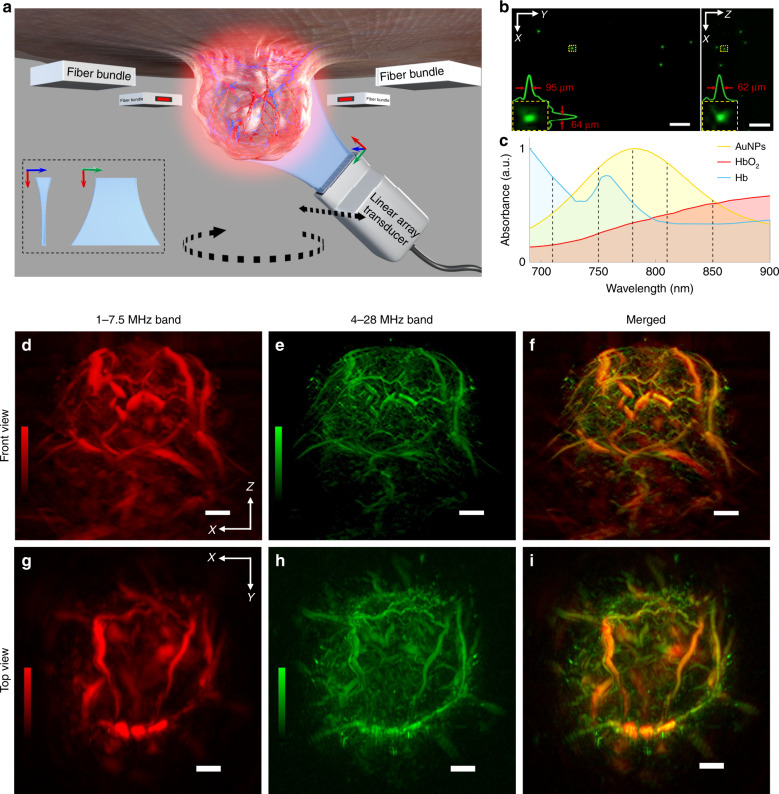
MSOM system for in vivo whole-tumour imaging and its basic characterization. **a** Schematic of the system. The entire solid tumour is illuminated from four sides by a four-arm fibre bundle. A cylindrically focused linear array is designed to detect optoacoustic signals from the tumour. In vivo imaging is performed in conical scanning geometry by controlling the rotation and translation stages. The sensing part of the transducer array and the tumour are submerged in water to provide acoustic coupling. **b** Maximum intensity projections of the optoacoustic reconstruction of a phantom of polyethylene microspheres (diameter, 20 μm) dispersed in agar. The inset shows a zoomed-in view of the region boxed with a yellow dashed line. In addition, the yellow boxes are signal profiles along the *x*, *y* and *z* axes across the microsphere centre, as well as the corresponding full width at half-maximum values. **c** Normalized absorption spectra of Hb, HbO_2_ and gold nanoparticles (AuNPs). The spectrum for the AuNPs was obtained using a USB4000 spectrometer (Ocean Optics, Dunedin, FL, USA), while the spectra for Hb and HbO_2_ were taken from http://omlc.org/spectra/haemoglobin/index.html. The vertical dashed lines indicate the five wavelengths used to stimulate the three absorbers: 710, 750, 780, 810 and 850 nm. Optoacoustic signals were filtered into a low-frequency band (red) and high-frequency band (green), which were used to reconstruct separate images. Maximum intensity projections from each frequency band are shown separately in the *xz* plane (**d**, **e**) and in the *xy* plane (**g**, **h**). **f** and **i** Merged images are shown in the right column. Scale bars, 1 mm

The MSOM system employed (Fig. 1a) is a second-generation mesoscopy setup with key innovations that allow in vivo visualization throughout entire solid tumours. The system uses four-sided illumination, and the generated acoustic signal is detected using a 96-element linear array detector with a central frequency of 15 MHz and bandwidth of ~1–30 MHz. This bandwidth was found to be optimal for imaging volumes measuring 5–10 mm in diameter, according to results from simulations and experimentation with other frequencies in the past^28–30^. A further novelty of the system is the combination of four-sided illumination and a conical geometry, in which the detector is positioned at a 45-degree angle to the rotation plane, thus allowing the accommodation of tumours of different sizes (Fig. 1a). MSOM imaging of tumours was performed at five wavelengths to capture the distributions of different intrinsic chromophores, such as deoxygenated haemoglobin (Hb) and oxygenated haemoglobin (HbO_2_), and of extrinsic probes such as gold nanoparticles (Fig. 1c).

Images were obtained by collecting data in translation-rotation conical scanning mode. Compared to rotation-only or translation-only scanning, translation-rotation scanning has previously demonstrated superior mesoscopic imaging performance^31^. Reconstruction was based on a backprojection algorithm^31^ adapted to the conical geometry. Previously reported methods for image reconstruction at different frequency bands and frequency equalization were borrowed from raster scan optoacoustics^32^ and applied for the first time in MSOM. We separated a low-frequency (1–7.5 MHz) and high-frequency (4–28 MHz) band (Fig. 1d–i). Band-specific reconstruction and frequency equalization have been shown to provide better signal-to-noise ratio and rendering than single-band reconstruction^32^, especially in regard to the high-spatial-frequency components (fine details) (SI Appendix, Fig. S1).

The resolution of the MSOM system was characterized using 20 μm polyethylene microspheres dispersed in an agar cylinder. The system resolved spheres with a full width at half-maximum diameter of 62 μm for full-frequency band reconstruction (Fig. 1b), indicating an in-plane system resolution of ~50 μm after deconvolution of the finite sphere size and total impulse response through at least ~1 cm of tissue (SI Appendix, Fig. S2a, d). The band-specific resolution was ~38 μm for the high-frequency band and ~92 μm for the low-frequency band, as also shown in SI Appendix, Fig. S2.

MSOM was used to interrogate differences in the spatial heterogeneity of total haemoglobin concentration (HbT = Hb + HbO_2_), oxygen saturation (sO_2_ = HbO_2_/HbT) and vascular permeability. HbT and sO_2_ were computed by resolving the distribution of Hb and HbO_2_ in entire tumours in vivo at resolutions never before possible. The spatial heterogeneity of these parameters was quantitated throughout the volumes of three types of breast tumours: two human breast cancer xenografts (KPL4, MDA-MB-231) and one mouse mammary tumour allograft (4T1). Vascular permeability in 4T1 tumours in mice following injection of gold nanoparticles was also studied.

**Fig. 2:**
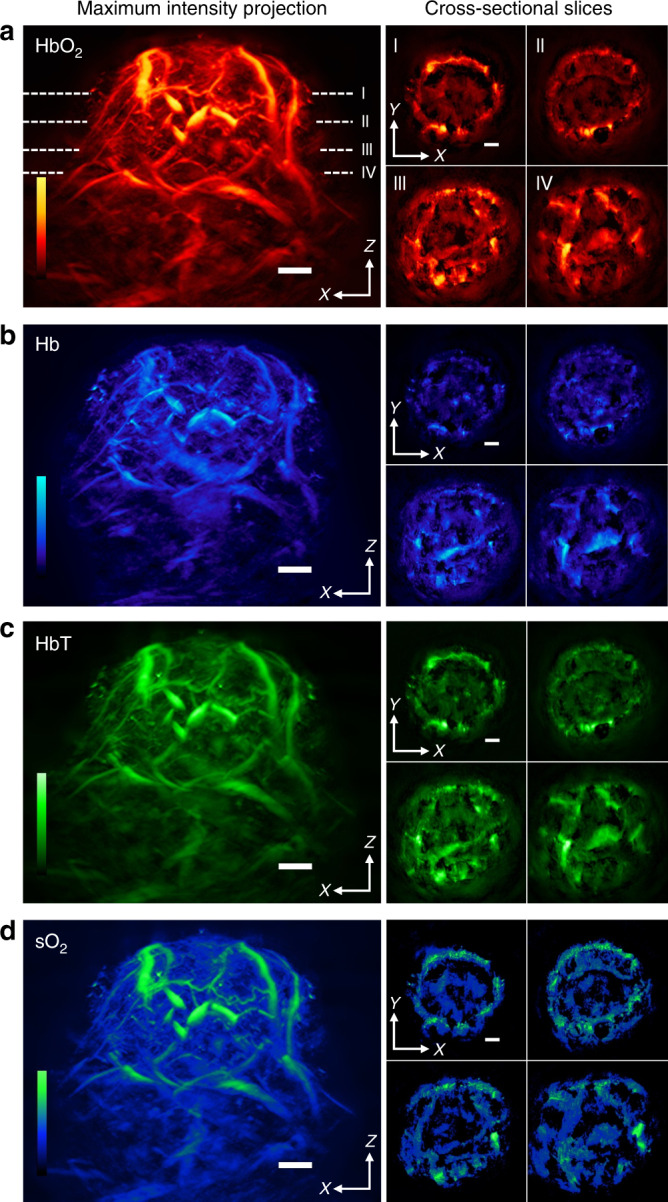
Whole-tumour in vivo MSOM imaging based on endogenous Hb and HbO_2_ contrast. Representative results are shown for one 4T1 tumour. The larger panels show maximum intensity projections in the *xz* plane for (**a**) HbO_2_, (**b**) Hb, (**c**) total haemoglobin concentration (HbT), and (**d**) oxygen saturation (sO_2_). Next to each larger panel are maximum intensity projections in the *xy* plane corresponding to the four tumour depths (I–IV). These four tumour sections compose the entire tumour depth. Maximum intensity projections were calculated over tumour sections with a thickness of 400 μm. Scale bars, 1 mm

Figure 2 shows the distribution of Hb, HbO_2_, HbT and sO_2_ throughout a 4T1 tumour with a diameter of 8 mm growing within the mouse mammary fat pad. The images demarcate the tumour boundaries and details of the vascular networks and heterogeneity patterns attributed to optical contrast throughout the tumour. Two tumour representations are shown. One depicts MIPs of the entire tumour in the axial dimension, as shown in the four large panels in Fig. 2. This representation provides a holistic view of the tumour volume and primarily reveals large peripheral feeder blood vessels surrounding the tumour surface, which appear to be connected with microvessels within the tumour. Two-band frequency equalization enables better separation of large vessels from smaller ones (see Methods and Fig. 1d–i). Extensive highly oxygenated areas are visible on the tumour periphery, while areas of lower oxygenation are visible in the tumour core. The second tumour representation, as shown in the smaller panels marked I–IV in Fig. 2, provides cross-sectional (coronal) views throughout the tumour mass. Four coronal slices of 400 μm thickness each are shown. This type of cross-sectional image showcases the unique abilities of MSOM, allowing the first observations of spatial patterns of HbT and sO_2_ within an entire tumour at resolutions previously inaccessible to in vivo optical imaging. Spectral error analysis highlights the high signal-to-noise ratio obtained in the images shown (SI Appendix, Fig. S3).

**Fig. 3:**
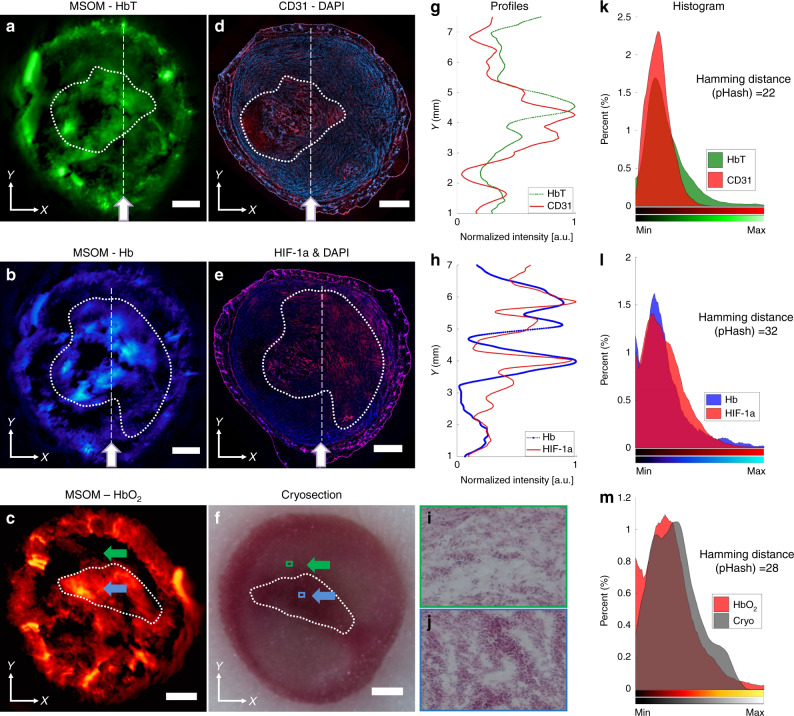
Validation based on histology and immunochemistry. **a**–**c** Maximum intensity projections of the distribution of total haemoglobin concentration (HbT), Hb and HbO_2_ in the *xy* plane of the 4T1 tumour close to the central plane. **d**–**f** Cryosections were taken at depths from within the region of the tumour used to generate these maximum intensity projections, and the sections were stained with haematoxylin-eosin, an antibody against endothelial marker CD31 or an antibody against hypoxia-associated HIF-1α. In some cases, the nuclear stain DAPI (blue) was also included. White dashed lines highlight regions with good correlation between optoacoustic images and histology micrographs. **g**, **h** Profiles were drawn along the straight white lines in (**a**) and (**d**) as well as (**b**) and (**e**). Green and blue arrows show the regions with opposite features in the HbO_2_ and HIF-1α images. **i**, **j** Higher magnification views of the cryosection regions boxed in green or blue are shown after staining with haematoxylin-eosin. **k**–**m** The histograms of optoacoustic and histological images in (**a**–**f**) are listed. Some images have been rotated to highlight correlations with other images. All maximum intensity projections in this figure were calculated over sections with a thickness of 400 μm. Scale bars, 1 mm

Tumours that were imaged in vivo were also processed ex vivo. The tumours were harvested from euthanized mice and preserved in tissue-freezing medium at −80 °C. Interleaved tumour tissue slices were stained with haematoxylin-eosin (H&E), anti-CD31 antibody for vascular endothelial cell staining^33^ and anti-HIF-1α antibody for identifying hypoxic areas in the tumour core^34^. The MSOM slices shown are of different thicknesses than the histology images. Moreover, the registration of MSOM images obtained in vivo and histological images obtained ex vivo with a different imaging system is only approximate. Nevertheless, comparison of MSOM slices and histological slices from approximately the same area/volume of the tumour (Fig. 3) revealed correspondence between the recorded signals. HbT-MSOM images corresponding to endothelial marker CD31 staining reveal the vasculature density, average radius (vessel size) and blood vessel distribution^25^ (Fig. 3a, d). Inspection revealed that the distribution of Hb correlated with immunohistochemical staining for hypoxia-associated HIF-1α (Fig. 3b, e). Profiles are obtained through images along the white lines (Fig. 3a, d) and then processed using the moving window averaging method. The profiles show similar variations in spatial patterns across the tumour (Fig. 3g, h) (see Methods). Overall, fluorescence micrographs show more abundant HIF-1α in the tumour core than in the middle region, consistent with the greater hypoxia in the core (SI Appendix, Fig. S4). Finally, we also compared MSOM images of the oxygenated haemoglobin distribution (Fig. 3c) to cryosections of the tumour (Fig. 3f) and H&E staining images of select regions (Fig. 3i, j). Figure 3f shows that the redder regions in the cryosection image corresponded to areas with increased oxygenated haemoglobin levels. The images in Fig. 3i, j are of H&E-stained microscopy slices taken from the areas indicated in Fig. 3c, f with the green and blue arrows, respectively. The H&E images show the different densities of tumour cells in regions of varying HbO_2_ concentrations. Figure 3k–m reveals further correspondence between the MSOM and histology results through a histogram and Hamming distance analysis of the images (see Methods). The Hamming distance was calculated by a perceptual image hash function (pHash) in MATLAB^35^. While the analysis in Fig. 3 is of slices of the 4T1 tumours, results obtained from KPL4 and MDA-MB-231 tumours were also validated histologically (SI Appendix, Figs. S5, 6).

**Fig. 4:**
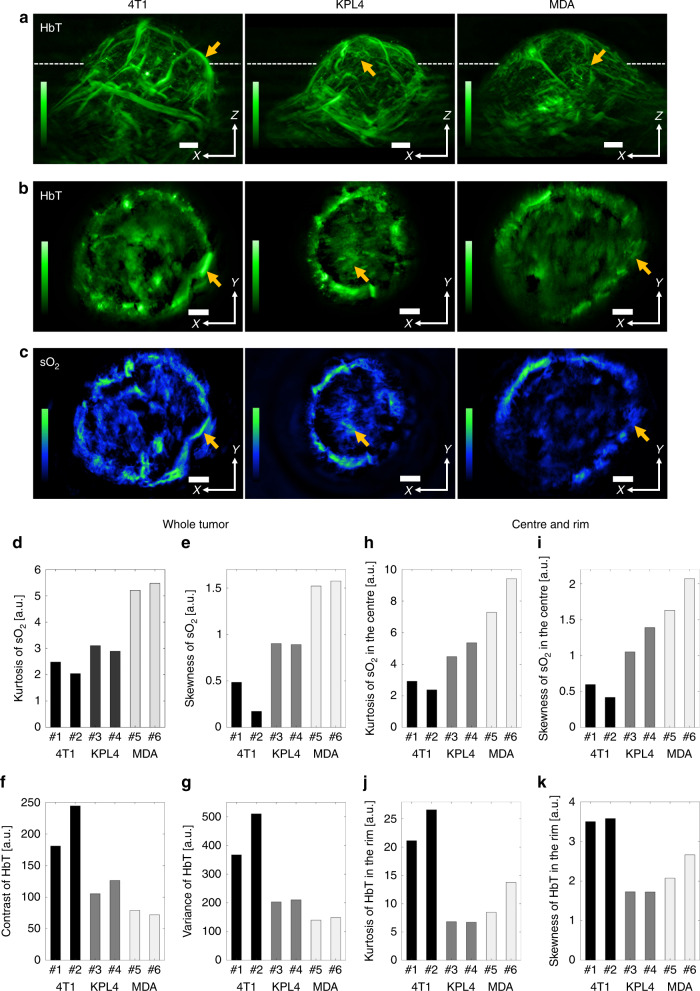
Comparison of in vivo MSOM images of three breast cancer tumour types and MSOM-based in vivo quantitation of sO_2_ and HbT heterogeneity throughout entire tumours. **a** The upper row shows representative maximum intensity projections of total haemoglobin concentration (HbT) in the *xz* plane of entire tumours in vivo. **b**, **c** Maximum intensity projections of the distribution of (**b**) HbT and (**c**) sO_2_ in the *xy* plane of the tumours, as defined by the dashed lines in panel (**a**). **d**–**k** Various heterogeneity metrics were measured in two biological replicates of the three types of breast cancer tumours. Details of how metrics were calculated can be found in the Methods section, together with an explanation of how tumours were segmented into centre and rim regions. MDA refers to MDA-MB-231. Some images have been rotated to highlight correlations with other images. All maximum intensity projections in this figure were calculated over sections with a thickness of 400 μm. Scale bars, 1 mm

Figure 4a–c extends the observations of tumour heterogeneity by comparing cross-sectional (coronal) images obtained throughout the tumour volume for all three tumour types (4T1, KPL4 and MDA-MB-231). These images allow a differential observation of HbT and sO_2_ patterns across different tumour types, pointing to the unique potential of MSOM to capture intra- and inter-tumour heterogeneity. Visual observation preliminarily revealed qualitative differences across tumours, with the MDA tumour exhibiting less optical contrast in its core and less superficial vasculature than the other tumours.

To quantitatively process and understand the heterogeneity patterns observed, we studied the Tamura metrics of coarseness, contrast and directionality^36^ as well as general metrics such as average intensity per region of interest, variance, skewness, kurtosis and energy based on histogram or statistical distributions^37^ (SI Appendix, Table S1). These traditional metrics have been previously applied to texture analysis of tumour heterogeneity using imaging modalities such as CT, MRI and PET^38–44^. For example, higher variance indicates greater dispersion around the average, corresponding to the presence of more highlighted objects with greater intensity differences from the background. Kurtosis indicates how irregular or flat the intensity histogram is relative to a Gaussian distribution. Kurtosis increases when pixel intensity is much brighter or darker than the background, but it decreases as the number of brighter or darker pixels increases^43^. This may make it an indicator of the vasculature complexity or necrosis degree. Skewness, which reflects the asymmetry of the histogram, refers to the average brightness of highlighted objects surrounded by background or less intense regions. Positive skew values indicate the presence of bright objects; negative skew values, the presence of dark objects. Contrast, which reflects variations between neighbouring pixels, relates to the dynamic range of intensity level in the image and may also indicate the extent of tumour heterogeneity. Variance, as an indicator of the number of dark or bright tumour regions corresponding to hypoxic areas or areas of high angiogenesis, may correlate directly with tumour heterogeneity. As shown in SI Appendix, Table S1, contrast is proportional to the square of variance, while kurtosis and skewness are inversely proportional to the third and fourth power of variance, respectively. In Fig. 4d–k, the metrics of both HbT and sO_2_ reflect the same correlations with their mathematical relations.

Figure 4d–g shows various metrics of sO_2_ and HbT heterogeneity on the whole-tumour level, while Fig. 4h–k shows metrics of heterogeneity separately for the tumour boundary (rim) and tumour core (centre). The results illustrate how MSOM can provide global and local quantitative functional information in a single non-invasive experiment. One intriguing result, for example, is that the KPL4 and MDA-MB-231 tumours showed higher sO_2_ kurtosis and skewness than 4T1 tumours (cf. panels d–e) and that this difference was even more apparent in the tumour centre than on the tumour rim (cf. panels h–i). Although the sO_2_ contrast and variance in 4T1 tumours were close to those in the two other tumour types, as shown in SI Appendix, Fig. S7c, d, the smaller differences in heterogeneity between the centre and rim in 4T1 tumours than in the tumours of the other types, as shown in SI Appendix, Fig. S7g, h, raises the possibility that 4T1 tumours are less heterogeneous in sO_2_ and less hypoxic overall than KPL4 or MDA-MB-231 tumours. SI Appendix, Fig. S8 shows the heterogeneity in HbT rather than sO_2_ for the three tumour types. As in the case of sO_2_, HbT heterogeneity varied substantially across tumour types. 4T1 tumours showed greater heterogeneity than the other two tumour types, and this heterogeneity may be present more in the rim than in the centre. These results provide the first evidence that MSOM can be employed to assess morphological and physiological differences within the tumour mass. Other heterogeneity metrics analysed at the whole-tumour level are listed in SI Appendix, Fig. S9. The MSOM-based in vivo quantitation of sO_2_ and HbT heterogeneity in individual *xy* plane sections of all tumour types and depths (SI appendix, Fig. S10) was comparable to that at the whole-tumour level (Fig. 4d–k).

**Fig. 5:**
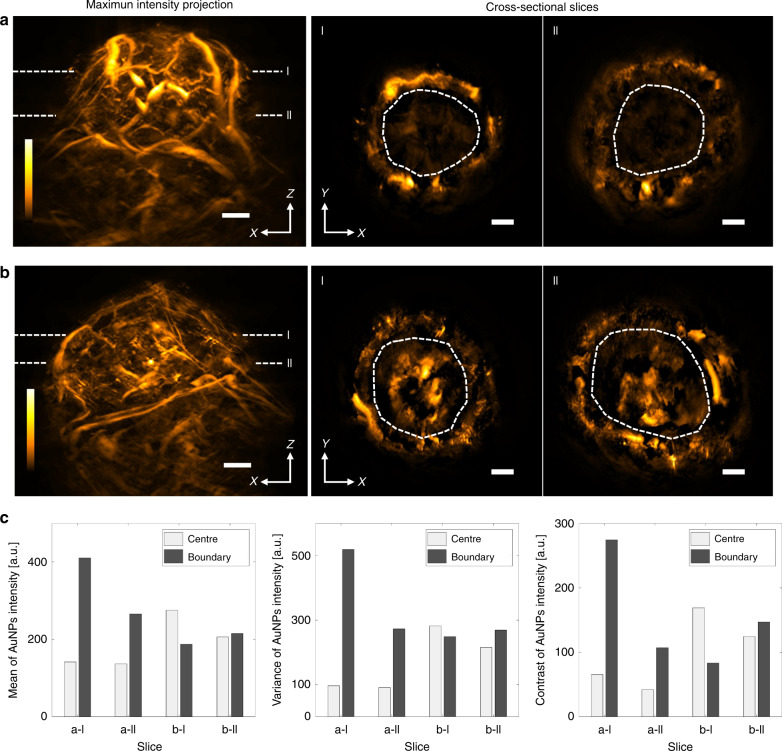
Whole-tumour in vivo MSOM imaging of nanoparticle perfusion. A mouse bearing a 4T1 tumour was injected with gold nanoparticles (AuNPs), and the animal was imaged at 1 and 24 h after injection. **a**, **b** Maximum intensity projections at (**a**) 1 h and (**b**) 24 h after injection. The left column shows projections in the *xz* plane, while the middle and right columns show projections in the *xy* plane at tumour depths I and II. Maximum intensity projections were calculated over sections with a thickness of 400 μm. **c** Quantitation of the mean AuNP intensity (left), variance in intensity (middle) and contrast of intensity (right) in the centre and boundary of the tumours in panels (**a**) and (**b**) at depths I and II. Dashed circles define the edge between the tumour centre and boundary. Scale bars, 1 mm

To explore the ability of MSOM to capture vascular permeability, we intravenously injected gold nanoparticles into a mouse bearing a 4T1 tumour. Such nanoparticles have been used for optoacoustic contrast enhancement and theranostics^45,46^. The mouse was imaged with MSOM throughout the entire tumour mass at 1 and 24 h after injection. Figure 5 shows that by 1 h, the optoacoustic signal due to the nanoparticles appeared primarily along the tumour boundary, and minimal signal was present within the tumour core; by 24 h, a substantial nanoparticle signal was present throughout the tumour core. Figure 5 shows nanoparticle distribution maps obtained for volumetric sections taken at two tumour depths, where the changes at different time points of perfusion progress can be obviously observed, especially in the comparison of various heterogeneity metrics between the centre and boundary of the tumour. Compared to the heterogeneity metrics at 1 h after injection, the metrics of the tumour centre approach or even exceed those of the tumour boundary at 24 h after injection. These results illustrate the ability of MSOM to track the penetration of nanoparticles as they move from the tumour periphery to the core. These results were validated by performing dark-field microscopy of tissue cryosections, in which a substantial gold nanoparticle signal was observed in the tumour core (SI Appendix, Fig. S11). SI Appendix, Fig. S12 shows the nanoparticle distribution in the three breast cancer tumour types at 24 h after injection. The 4T1 tumour shows higher nanoparticle intensity in the centre than do the other two tumour types. The kurtosis, skewness, contrast and variance metrics varied across the three tumour types, especially when we took the ratio of the tumour centre to the tumour boundary. The four metrics show similar values at the centre and boundary in the case of the MDA tumour but different values at the centre and boundary in the case of the 4T1 and KPL4 tumours. In fact, the two tumour regions differ in opposite directions between 4T1 and KPL4 tumours.

## Discussion

For the first time, we characterized the spatial heterogeneity of tumour hypoxia (sO_2_) and the corresponding vascular patterns throughout entire tumours in vivo at resolutions never before demonstrated by another method. By doing so, we also demonstrated that by using optoacoustic imaging, we can observe different patterns between tumours, allowing study of their individualized morphology and functions.

Focal hypoxia of solid tumours has been considered a prognostic biomarker and driver of tumour progression because of its connection with poor patient outcomes and therapy resistance^1^. Therefore, quantitating hypoxia in this way may provide valuable information for screening drug candidates and assessing patient response to therapy^19^. Segmented quantitation of HbT further allowed us to examine vascularization in different parts of the tumour, demonstrating divergent heterogeneous patterns. Such analyses of sO_2_ and HbT, which can assess the hypoxic microenvironment and vessel abnormalities of solid tumours, may provide a platform for the in vivo assessment of emerging anticancer therapies aimed at alleviating hypoxia and inhibiting tumour angiogenesis^1,47^. Poor tumour oxygenation has been linked to treatment resistance and worse outcomes^19^.

Overall, this study revealed that significant changes in optical contrast exist in different parts of the tumour. In particular, the tumour boundary showed angiogenesis and oxygenation patterns that differ significantly from those in the tumour core. However, this heterogeneity is not entirely predictable: for example, the tumour core also contains foci of vascularization and oxygenation intermingled with areas of severe hypoxia. For this reason, intravital microscopy, although it reveals sub-micrometre details of tumour biology, can provide only a local, fragmented understanding of tumour structure and function. MSOM, by covering a volume three orders of magnitude larger than intravital microscopy (~50 vs. 0.5 μm), provides a holistic view of tumours. In this way, the two techniques are highly complementary: MSOM can provide an overall view, within which specific regions can be analysed at higher resolution using intravital microscopy.

The results of this pilot study on technology feasibility clearly demonstrated differences in the heterogeneity present in different tumours. The study shows that the novel 15 MHz MSOM system can indeed interrogate the variability between tumour types and explore the relation between tumour type and heterogeneity in morphological and functional parameters such as vascularization and sO_2_ on a tumour-to-tumour basis. The relatively high quality of the images provided by the MSOM system described here likely reflects critical improvements over our previously published design^28^. The linear transducer array with a central frequency of 15 MHz can capture acoustic signals at a high signal-to-noise ratio from light-absorbing anatomical structures in the tumour smaller than 100 μm (blood vessels) up to structures spanning several millimetres (tumour core). The tumour in our system was illuminated with broad-field illumination from four sides, helping provide homogeneous stimulation of light absorbers in the tumour, leading in turn to stronger and more uniform acoustic signals despite depth-dependent attenuation of light fluence. The custom-designed sample holder features a tumour hole with plastic snap rings of various inner diameters that can be optimized to the tumour size. In addition, a linear stage allows the sample holder to be raised or lowered to position the sample optimally within the field of view of the detector. Frequency equalization, in which the relative contributions of acoustic signals of different frequencies are balanced, improves image fidelity by recovering chromophores of various sizes in the tumour, such as smaller and larger vessels. This advanced performance was essential to provide a detailed analysis of and insights into tumour patterns.

Our pilot study suggests that 4T1 may be less heterogeneous in sO_2_ and less hypoxic than KPL4 or MDA-MB-231 tumours; nevertheless, as a future step, it would be interesting to investigate these findings in a larger tumour sample in the context of explaining the particularly aggressive growth and multi-site metastasis of 4T1 tumours^48^. Tumour physiological heterogeneity and, in particular, oxygenation parameters can only be resolved at resolution orders of magnitude lower using nuclear imaging techniques, which require radioactivity and thereby limit possibilities for longitudinal studies. The possibility of using high-resolution MSOM to analyse tumour heterogeneity may create new opportunities for studying tumour heterogeneity in a more detailed manner. Indeed, MSOM fulfils one set of proposed requirements^49^ for analysing three-dimensional variations in tumour properties: high spatial resolution (10–100 μm) and a unique contrast and feature set not available to other imaging modalities. The study suggests the potential of textural measures of spatial heterogeneity such as skewness and kurtosis for image-based analysis of breast cancer. Skewness describes tumour characteristics, and in our samples, more extensive angiogenesis (4T1) was associated with greater positive skew values than less extensive angiogenesis (MDA and KPL4). The kurtosis values in our samples were inversely proportional to noise. The contrast and variance in our samples correlated negatively with hypoxia but positively with angiogenesis, which can be seen in the sO_2_ difference between the tumour centre and rim and in the HbT values at the rim. Measures of heterogeneity such as kurtosis are employed in other imaging modalities, such as X-ray CT^44,50^, PET^39,50^ or MRI^41^, for assessing tumour malignancy or staging^51–56^, therapeutic response and efficacy^41,57^ and survival outcomes^58–61^. In this study, we employed these metrics for the first time in analysing optical contrast in high resolution, showcasing previously undisclosed optical and sO_2_ variability between the tumours studied. Compared to earlier work on tissue heterogeneity, such as spectrum analysis approaches^62–64^ and MSOT^18^, our work focused on providing a mesoscopic tool operating at a centre frequency of 15 MHz to offer in vivo three-dimensional functional images with resolutions of 38–92 μm throughout whole solid tumours. Therefore, MSOM allows us to observe fine details of cancer haemodynamic contrast that go beyond the intensity and frequency signals of optoacoustic images obtained at macroscopic resolutions.

Uniquely among high-resolution imaging techniques, MSOM can visualize tissue features based on endogenous contrast from Hb, HbO_2_, lipids and melanin, as well as exogenous contrast based on nonspecific dyes and specific targeting agents^46,65^, at high resolution. Spectral unmixing allows the contributions of these various contrast agents to be separated, enabling the simultaneous imaging of different markers of health and disease. In the present work, we demonstrate the simultaneous imaging of vasculature and gold nanoparticles at the level of entire tumours. This raises intriguing possibilities for tumour analysis that aims to correlate, for example, oxygenation and the activity of specific subsets of tumour and host cells, such as tumour-associated macrophages, which can be tracked in vivo using specifically designed nanoparticles^66^. Perfusion of gold nanoparticles into tumours could be accessed longitudinally in a non-invasive manner at a level of intra-tumoural detail not previously achieved with other tomographic set-ups^18,19,67^ and with a whole-tumour imaging volume inaccessible to intravital microscopy. This finding highlights the potential of MSOM for preclinical screening and assessment of anticancer therapeutics in vivo or in organoids. The technique’s combination of penetration depth, resolution, whole-tumour imaging, and non-invasiveness may give it advantages over intravital microscopy as a key tool in the drug discovery process^68,69^, such as for clarifying why nanoparticle-based cancer treatment often fails to improve therapeutic outcomes^70^.

Future efforts to improve the current MSOM system should focus on reducing measurement time using lasers with high repetition rates and fast wavelength scanning. Future work will introduce more image analysis methods to improve the quantitative performance of MSOM in tumour heterogeneity analysis.

Overall, MSOM is shown to image entire tumour morphology and physiology in vivo in a label-free, non-invasive manner at depths and resolutions never before demonstrated with an optical or optoacoustic method. By using a transducer array at a 15 MHz central frequency and applying frequency equalization, our method allowed unprecedented cross-sectional imaging and achieved resolutions never before achieved in whole-tumour imaging by an optical contrast imaging method. As a result, we were able to quantify patterns of heterogeneity at the entire-tumour level and differentially quantify patterns of heterogeneity within parts of the tumour, such as the core and the rim. With its demonstrated abilities and the potential for hardware and software improvement, MSOM may prove to be a uniquely powerful preclinical technology on the road towards precision oncology, which aims to characterize tumour heterogeneity in order to select the most appropriate therapies and optimize prognosis.

## Materials and methods

### In vivo tumour models

Animal procedures were approved by the Government of Upper Bavaria. Female athymic *Foxn1* nude mice (5-week old, Envigo, Germany) were injected into the mammary fat pad with 4T1 murine breast cancer cells (CRL-2539, ATCC, Manassas, VA, USA; 1 × 10^4^ cells/animal) or KPL4 human breast cancer cells^71^ (kindly provided by J. Kurebayashi, Kawasaki Medical School, Kurashiki, Japan; 2 × 10^6^ cells/animal). NOD/SCIDSHrN nude mice (5-week old, Charles River Laboratories, Germany) were injected into the mammary fat pad with MDA-MB-231 human breast cancer cells (CRM-HTB-26, ATCC; 3 × 10^6^ cells/animal). Each type of cancer cell was injected into three animals under isoflurane anaesthesia. When tumours reached a diameter of ~8 mm, the animals were anaesthetized and imaged using MSOM. This tumour size was reached within 7–10 days in 4T1-injected animals, within approximately 40 days in KPL4-injected animals, and in 30–40 days in MDA-MB-231-injected animals. At this time, a mouse carrying a 4T1 tumour was injected intravenously with 50 μl of gold nanoparticles (D12M-780–50, Nanopartz, USA) suspended in 200 μl of phosphate-buffered saline (PBS) and then imaged in vivo.

### MSOM setup

Figure 1a shows the custom-built MSOM system used for all experiments. The system features a conical scanning geometry for in vivo tumour imaging^28,31,72,73^. Single motorized stages for rotation (M-062. PD, Physik Instrumente, Germany) and translation (M-605.2DD, Physik Instrumente) allow scanning in discrete or continuous modes^31^. In the present study, in vivo imaging was performed in continuous scanning mode with an angle range of 255° and a translation range of 10 mm. Samples were excited optically using an optical parametric oscillator laser (Phocus II, Opotek, USA) tunable from 690 to 900 nm and capable of providing sub-10 ns pulses at a 10 Hz repetition rate and maximum pulse energy of 80 mJ at 760 nm. The laser beam was delivered to the sample using a custom-made bundle of 672 fibres (Ceramoptec, Germany) divided into four arms, each of which measured 6.5 × 2.5 mm and was positioned on a different side of the sample. This setup allowed homogeneous illumination of a volume of approximately 14 × 14 × 6 mm. Laser pulse energies were selected based on sample size and were always between 30 and 50 mJ to remain below the ANSI-defined limit for biological samples. The average laser power was measured before each experiment at each wavelength using a Vega power metre (Ophir Photonics, Israel) to allow light fluence correction.

A sample holder was custom-designed to accommodate an entire mouse as well as the four heads of the fibre bundle, which were positioned at an angle of 8° from the *xy* plane. The holder was attached to a linear stage (NRT150, Thorlabs, Newton, NJ, USA) to allow adjustment of the holder position along the *z* axis. The anaesthetized mouse was placed on top of the holder such that the tumour protruded through a hole and into a water bath held at 33 °C. The fibre bundle heads and transducer array were also immersed in the water bath. This bath provided acoustic coupling between the tumour and the transducer array. Snap rings with the same outer diameter and different inner diameters were fitted onto the hole according to tumour size in order to optimize sample placement and minimize the generation of acoustic signals from neighbouring tissues.

The ultrasound response from the sample was detected using a custom-made linear transducer array (Vermon, France) with 96 elements, a 15 MHz central frequency and an average −6 dB pulse-echo bandwidth of 45%. Each element has a pitch of 100 μm, an elevation of 1.5 mm and a cylindrically focused feature with a focal length of 7.8 mm. The array is mounted at 45° on a custom-made tilting module connected to the translation-rotation scanner, allowing conical scanning around the tumour sample. The radius of the central plane traced by the central (48th) element in the array was 5.8 mm. The linear array rotated and translated continuously at speeds chosen to provide optimal sample coverage at the 10 Hz repetition rate of the laser. Between two successive laser pulses, the array rotated by ~0.035° and translated ~200 nm.

### Calibration of the MSOM system

The MSOM system was used to visualize a phantom consisting of black polyethylene microspheres with a diameter of 20 μm (BKPMS 20–27, Cospheric, USA) uniformly dispersed in agar gel cylinders with a diameter of 12 mm. These absorbers generate broadband optoacoustic signals with a frequency band wider than the maximum bandwidth of the linear array for the given laser pulse duration. The data collected with this phantom were used to calibrate the MSOM set-up, to measure the average speed of sound and to determine the spatial resolution of the system.

### Data acquisition and image reconstruction

Approximately 7000 × 96 optoacoustic signals were acquired at each wavelength and digitized using a custom-built data acquisition card (Falkenstein, Germany) operating at 10 Hz and sampling at 125 MS/s with 12-bit resolution over a 16 mV range. The total scanning time per wavelength was 12 min.

Ultrasound data collected after excitation at five wavelengths were used for tumour image reconstruction (Fig. 1c). Raw data were bandpass-filtered using a third-order Butterworth filter (1–28 MHz) to remove noise and then passed through a time-variant filter^74^ to compensate for frequency-dependent acoustic attenuation. Three-dimensional images were reconstructed using a backprojection algorithm^75^ with a voxel size of 24 × 24 × 24 μm. Reconstructed images were corrected for light fluence as previously described^76^. Frequency equalization was performed by reconstructing optoacoustic images in two frequency bands of 1–7.5 MHz and 4–28 MHz, and the image features from each band were coloured differently and overlaid using ImageJ^77^. Finally, linear spectral unmixing^29,78^ was used to generate separate reconstructions of the distribution of endogenous or exogenous contrast agents. The unmixing algorithm assumed a constant background spectrum and absorption spectra of Hb, HbO_2_, and gold nanoparticles (Fig. 1c). Unmixed images were generated and analysed using ImageJ. For display purposes, the gamma function in ImageJ was used in some figures to highlight information from the tumour core, but all quantitative analyses were performed on unmixed results without further processing. The unmixing algorithm provides values (Hb, HbO_2_ and gold nanoparticles) that are in arbitrary units and relative across different structures in the reconstructed images. The sO_2_ values are relative rather than absolute, as they are calculated as ratios from the Hb and HbO_2_ values.

### Tumour heterogeneity analysis and tumour segmentation

The presented metrics were calculated by averaging the metric values in MIPs of 400 μm sections at different tumour depths throughout the entire tumour. Variance, skewness, kurtosis, energy and entropy were calculated as previously described^37^. Coarseness, contrast and directionality were calculated using Tamura texture analysis^36^. The centre and rim regions of tumours were partitioned using cut-off points identified from the lines connecting the centre point and boundary points. Relative differences were calculated as the ratio between a metric’s absolute difference between the centre and rim to the metric’s sum for the centre and rim.

### Spectral error maps

We assessed the reliability of MSOM results by calculating fitting residuals describing the pixel-wise deviations between the reconstructed multi-spectral image and the corresponding linear unmixed signal. The MIP across 20 slices of the signal norm was computed, and each pixel of the resulting multispectral image was fitted to the spectra of Hb and HbO_2_ or gold nanoparticles. Then, the norm of the residuals relative to the norm of the signal was displayed as an image to identify the relative uncertainty associated with different parts of the tumour image (SI Appendix, Fig. S3).

### Tumour histology, immunohistochemistry, and lectin staining

Cryosections (10 µm thick) were prepared using standard procedures (CM1950Cryostat, Leica). For haematoxylin-eosin staining, sections were thawed, dried on a heating plate, fixed in paraformaldehyde and stained with haematoxylin (30 s) and eosin (1 s) using standard procedures. For immunohistochemistry, sections were dried on a heating plate, fixed in cold acetone for 10 min, and then rinsed in Tris-buffered saline containing 0.3% Triton X-100 (TBST). Slices were blocked for 1 h at room temperature with TBST containing 10% goat serum. The blocking solution was removed, and sections were immunostained overnight at 4 °C with a rat primary antibody against mouse CD31 (1:20; DIA-310, Dianova, Hamburg, Germany) to show the blood vessel distribution. Sections were washed three times (10 min each) with TBST, incubated for 1 h at room temperature with an Alexa Fluor 594-conjugated goat anti-rat secondary antibody (1:200; A-11007, Thermo Fisher Scientific, Munich, Germany), and then washed again three times (10 min each) with TBST. Alternatively, sections were immunostained against HIF-1α as described above except using a rabbit primary antibody against mouse HIF-1α (1:50; ab2185, Abcam, Cambridge, UK) and an Alexa Fluor 488-conjugated goat anti-rabbit secondary antibody (1:200; A-11034, Thermo Fisher Scientific). All antibodies were diluted in TBST containing 1% goat serum. Sections were mounted using ProLong Gold Antifade Reagent with DAPI (catalogue no. 8961S, Cell Signalling Technology, MA, USA) and analysed using a Zeiss Axio Imager M2 microscope and Zeiss ZEN 2 Pro (Blue edition) software.

In some experiments, animals were injected intravenously with pimonidazole-HCl (80 mg/kg) from the Hypoxyprobe Kit (Hypoxyprobe, Burlington, MA, USA) and sacrificed 1 h later. Pimonidazole distributes to all tissues and forms adducts with thiol-containing proteins in cells where the oxygen partial pressure is below approximately 10 mmHg at 37 °C^79–81^. Tumour sections were prepared and immunostained as described above using the mouse primary monoclonal antibody supplied in the Hypoxyprobe Kit and an Alexa Fluor 594-conjugated goat anti-mouse secondary antibody (1:200; A-11005, Thermo Fisher Scientific).

### Profile, histogram and Hamming distance

Profiles in Fig. 3g, h were obtained along the white lines indicated in Fig. 3a, d and Fig. 3b, e. These line profiles were smoothed by the moving mean function (in MATLAB) with window lengths of 60 and 240. The histogram with a total of 256 bins was constructed in the undersampled images of 256 × 256 pixels with background subtraction. Undersampling was performed using spline interpolation in Fig. 3a–f.

Based on an image’s visual appearance, a discrete cosine transform (DCT)-based image hashing algorithm can produce a hash value for each image stored in an unsigned 64-bit integer^35^. Hamming distance can be used to measure the differences between two hash values within the range from 0 to 64 and then identify whether two images are perceptually different or similar. The maximum Hamming distance of 32 in Fig. 3k–m indicates that at most half of the elements in the two hash values are different. If a pre-defined threshold is set to 32.00 and if both images have Hamming distances smaller than this threshold in pHash, then the two images can be considered perceptually similar.

## Supplementary information

Supplementary Information
