# Supplementary material for: Spatial heterogeneity of oxygenation and haemodynamics in breast cancer resolved in vivo by conical multispectral optoacoustic mesoscopy
Source: Light Sci Appl. 2020 Apr 13;9:57. doi: 10.1038/s41377-020-0295-y (PMC7154032; doi:10.1038/s41377-020-0295-y)
Supplement: Supplementary file 1 — Supplementary Information [file 41377_2020_295_MOESM1_ESM.docx]

Supplementary Information for

**Spatial heterogeneity of oxygenation and haemodynamics in breast cancer resolved *in vivo* by conical multispectral optoacoustic mesoscopy**

Jiao Li, Andrei Chekkoury, Jaya Prakash, Sarah Glasl, Paul Vetschera, Benno Koberstein-Schwarz, Ivan Olefir, Vipul Gujrati, Murad Omar, Vasilis Ntziachristos *

*Corresponding to: [v.ntziachristos@tum.de](mailto:v.ntziachristos@tum.de) (V.N.)

**Supplementary Information includes:**

- **Supplementary text**: Results on **Fig. S7**
- **Supplementary Table**: Mathematical expressions of metrics
- **Supplementary Figure**: Figs. S1 to S12
- **References**

**Supplementary Text**

**Results on** **Fig. S7**

**Fig. S7 a-d** shows various metrics of sO_2_ heterogeneity on the whole-tumour level, while **Fig. S7 e-h** shows metrics of heterogeneity separately for the tumour boundary and tumour core (centre). The results illustrate how MSOM can provide global and local quantitative functional information in a single non-invasive experiment. One intriguing result, for example, is that the KPL4 and MDA-MB-231 tumours showed higher sO_2_ kurtosis and skewness than 4T1 tumours (cf. panels a-b), and this difference was even more apparent in the tumour centre (cf. panels e-f). Although sO_2_ contrast and variance in 4T1 tumours were close to those of the two other tumour types (cf. panels c-d), the smaller differences in heterogeneity between centre and rim in 4T1 tumours than in tumours of the other types (panels g-h) raises the possibility that 4T1 tumours are less heterogeneous in sO_2_ and less hypoxic overall than KPL4 or MDA-MB-231 tumours.

**Supplementary Table**

**Table S1. Mathematical expressions of metrics^1, 2^**

| Metric name | Mathematical expression | Metric name | Mathematical expression |
| --- | --- | --- | --- |
| Average | $\mu=\frac{1}{MN}\sum_{i=1}^{M} \sum_{j=1}^{N} p\left(i,j\right)$ | Variance | $\sigma=\sqrt{\frac{1}{MN}\sum_{i=1}^{M} \sum_{j=1}^{N} \left(p\left(i,j\right)-\mu\right)^{2}}$ |
| Kurtosis | $\frac{1}{MN}\sum_{i=1}^{M} \sum_{j=1}^{N} \left[\frac{p\left(i,j\right)-\mu}{\sigma}\right]^{3}$ | Skewness | $\frac{1}{MN}\sum_{i=1}^{M} \sum_{j=1}^{N} \left[\frac{p\left(i,j\right)-\mu}{\sigma}\right]^{4}$ |
| Energy | $\sum_{L=1}^{255} \frac{L\times n_{L}}{MN}$ | Contrast | $\frac{\sigma}{\alpha_{4}^{1/4}}$ |
| Coarseness | $\frac{1}{MN}\sum_{i=1}^{M} \sum_{j=1}^{N} S_{\text{best}}\left(i,j\right)$ | Directionality |  |

In **Table S1**, image size is $M\times N$ ($i=1,\cdots,M$ and $j=1,\cdots,N$). $p\left(i,j\right)$ is pixel value. $n_{L}$is the component of histogram. $\alpha_{4}=\mu_{4}/\sigma^{4}$. For Coarseness^2^, the average over the neighborhood of size $2^{k}\times2^{k}$at the point $\left(x,y\right)$ is $A_{k}\left(x,y\right)=\sum_{i=x-2^{k-1}}^{x+2^{k-1}-1} \sum_{j=y-2^{k-1}}^{y+2^{k-1}-1} f\left(i,j\right)/2^{2k}$, where $f\left(i,j\right)$is the grey-level at $\left(x,y\right)$. The difference in the horizontal case is $E_{k,h}\left(x,y\right)=\left|A_{k}\left(x+2^{k-1},y\right)-\right.$ $\left.A_{k}\left(x-2^{k-1},y\right)\right|$. The best size with the highest output value is $S_{\text{best}}\left(i,j\right)=2^{k}$. $k$ maximizes $E$ in either direction, i.e., $E_{k}=E_{max}=max\left(E_{1},E_{2},L,E_{L}\right)$. For directionality^2^, $n_{p}$ is number of peaks; is the *p*^th^ peak position of $H_{D}$; $w_{p}$ is range of the *p*^th^ peak between valleys; $r$ is normalizing factor related to quantizing levels of ; is quantized direction code (cyclically in modulo 180º).

$H_{D}\left(k\right)=N_{\theta}\left(k\right)/\sum_{i=0}^{n-1} N_{\theta}\left(i\right)$, $k=0,1,\cdots,n-1$

where $N_{\theta}\left(k\right)$is the number of points at which $\left(2k-1\right)\pi/32\leq\theta<\left(2k+1\right)\pi/32$and $\left|\Delta G\right|\geq12$.

The magnitude $\left|\Delta G\right|$ and the local edge direction $\theta$ are approximated as follows:

$$\left|\Delta G\right|=\left(\left|\Delta_{H}\right|+\left|\Delta_{V}\right|\right)/2$$

$$\theta=\tan^{-1}\left(\Delta_{V}/\Delta_{H}\right)+\pi/2$$

**Supplementary Figure**


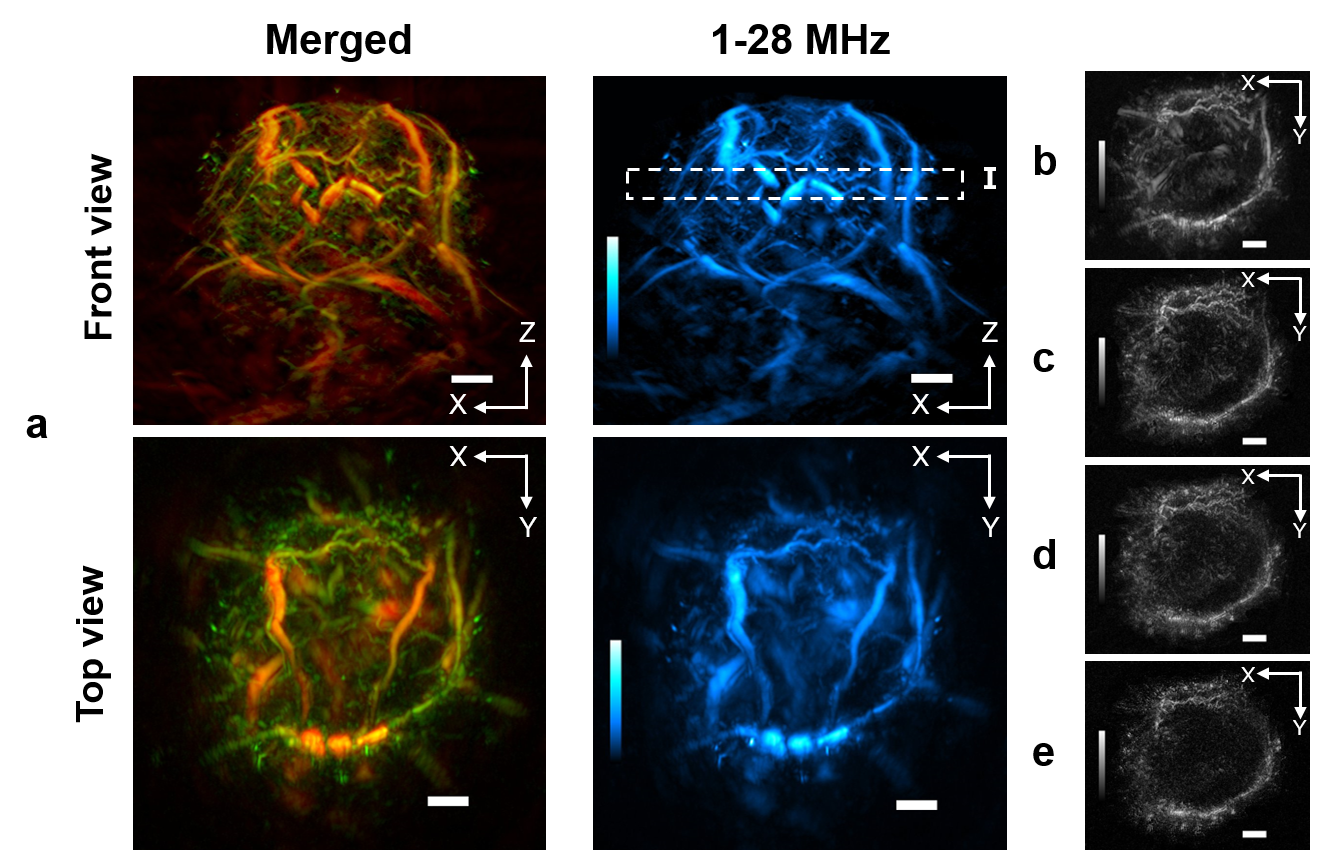


**Fig. S1.** **Comparison of merged image reconstructions and reconstructions based on different frequency bands.** **(a)** The merged images are the same as those in **Fig. 1f, i**. Maximum intensity projections of 1-28 MHz are shown separately in the *xz* plane (front view) and in the *xy* plane (top view). **(b-e)** Cryosections were taken at depth I from within the tumour regions used to calculate the maximum intensity projections in the *xy* plane with different frequency bands of **(b)** 4-28 MHz, **(c)** 10-28 MHz, **(d)** 15-28 MHz and **(e)** 20-28 MHz. Scale bars, 1 mm.


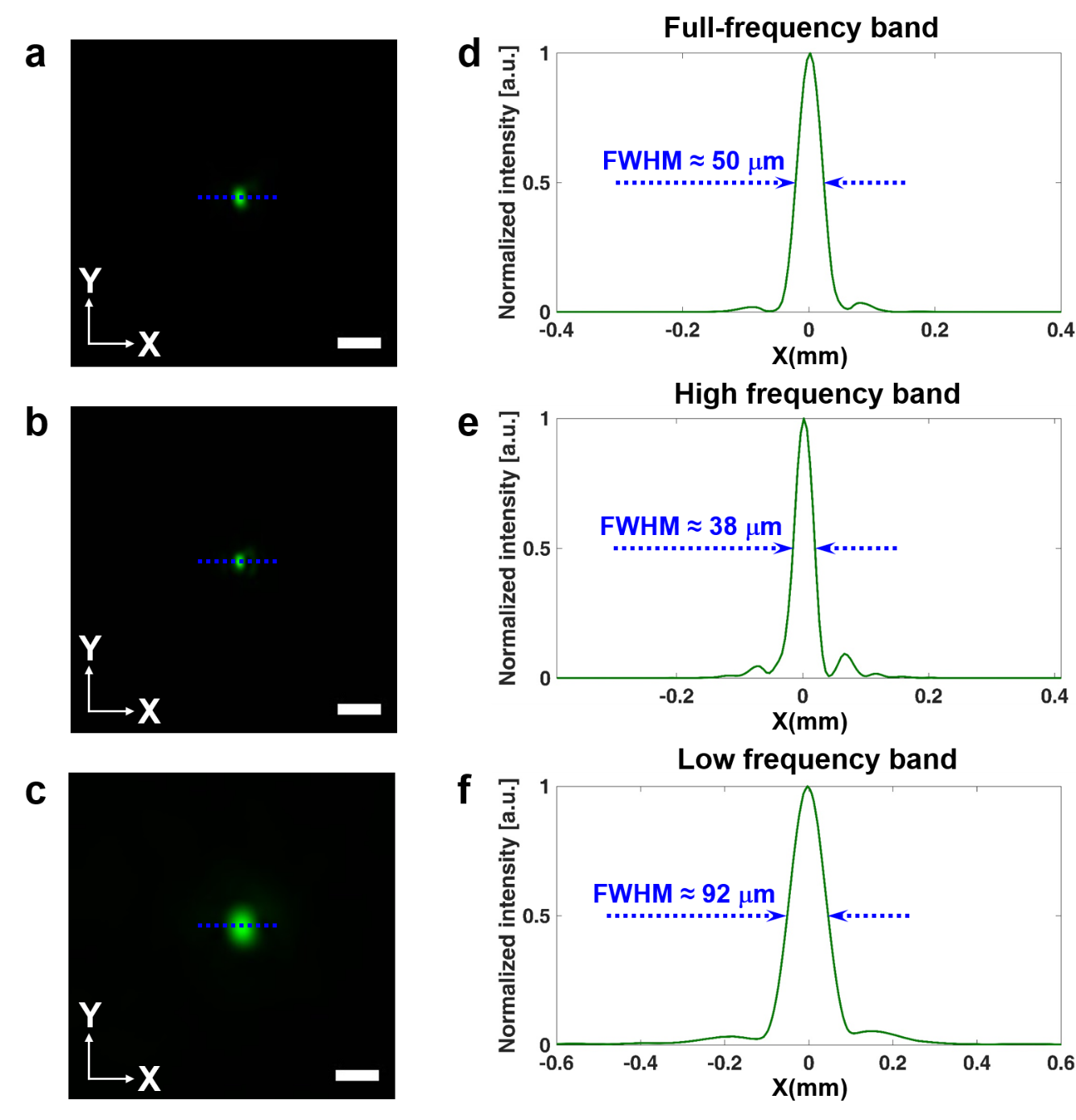


**Fig. S2. Measurement of band-specific resolution for different frequency bands.** Maximum intensity projections of the optoacoustic reconstruction of a phantom embedding a 20 μm polyethylene microsphere using optoacoustic signals from frequency bands of **(a)** 1-28 MHz, **(b)** 4-28 MHz and **(c)** 1-7.5 MHz. The optoacoustic images are recovered after deconvolution of both the finite sphere size and total impulse response. **(d-f)** Their profiles along the blue dashed lines across the microsphere centre, as well as the corresponding values of full width at half-maximum. Scale bars, 200 μm.

**
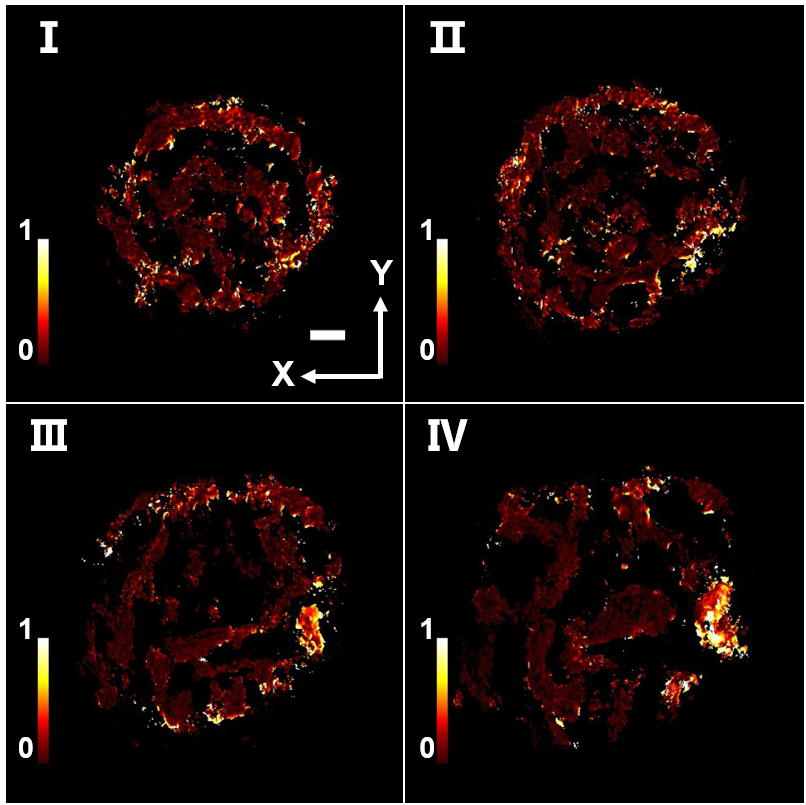
**

**Fig. S3. Spectral error in optoacoustic imaging of tumours *in vivo*.** Uncertainty associated with optoacoustic signal fitted to each voxel within the imaged region was assessed by calculating fitting residuals and examining their distribution in the tumour. The four images show representative results for a 4T1 tumour after 14 days of growth at different depths I-IV; this tumour is the same as the one shown in **Fig. 2**. The normalized fitting residuals at each voxel were then displayed as an image. Voxels with an optoacoustic signal intensity <2% of the maximum in the 20 slices were shown with a black background to allow more sensitive noise detection, since noise should disproportionately affect voxels of extremely low signal intensity. In addition, voxels with negative signal intensity were shown with a black background since the spectral unmixing algorithm^3^ ignores negative signals (see Methods). Scale bar, 1 mm

**
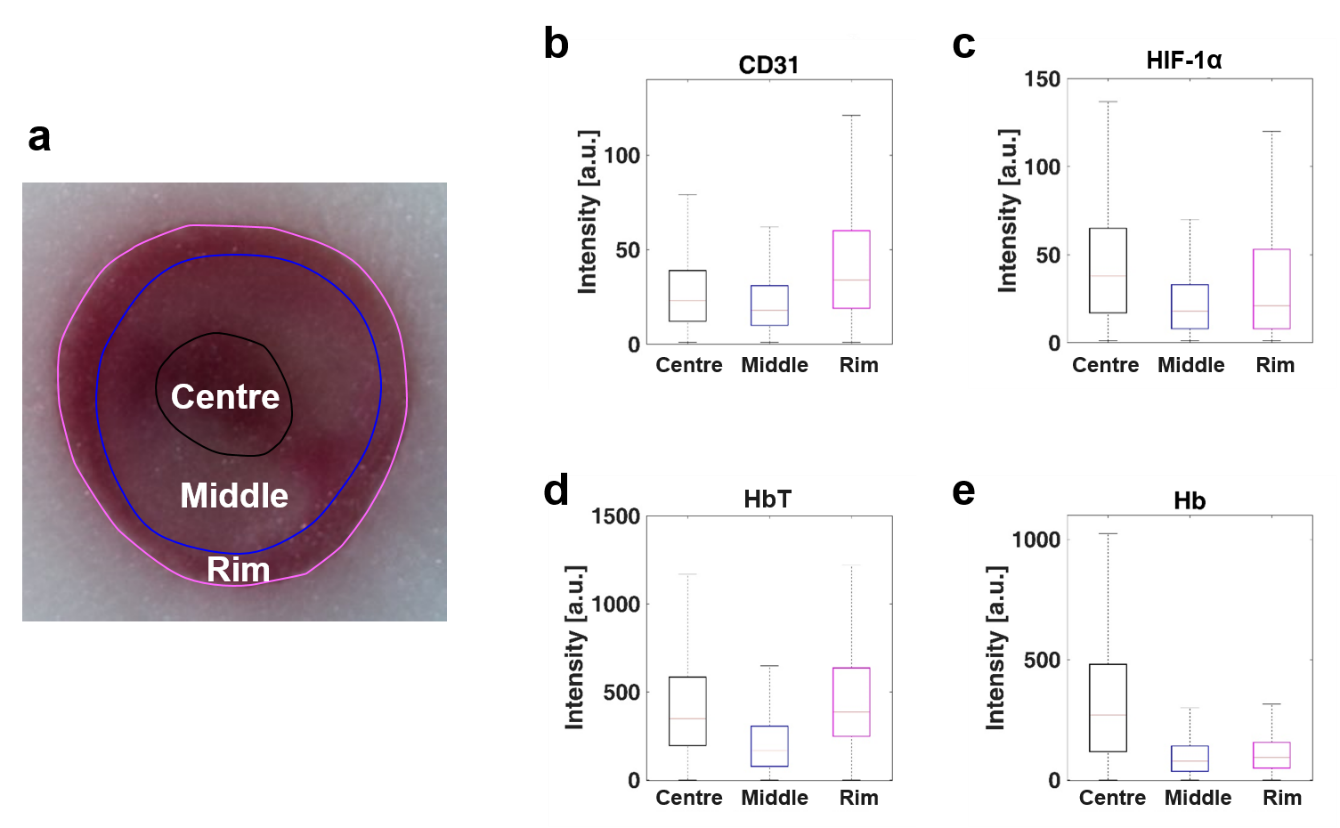
**

**Fig. S4. Validation of whole-tumour MSOM measurements against histology and immunochemistry of cryosections (4T1).** The same 4T1 tumour shown in Fig. 3 was segmented into centre, middle and rim regions as shown in panel **(a),** and the corresponding regions of the stained cryosections shown in **Fig. 3f** were quantitated. HbT based on the MSOM signal in panel **(d)** was slightly higher at the tumour rim than in the centre, consistent with vessel distribution based on CD31 staining in panel **(b).** Hypoxia, based on anti-HIF-1 staining in panel **(c),** was slightly greater in the centre than at the rim, consistent with Hb distribution based on the MSOM signal in panel **(e).**


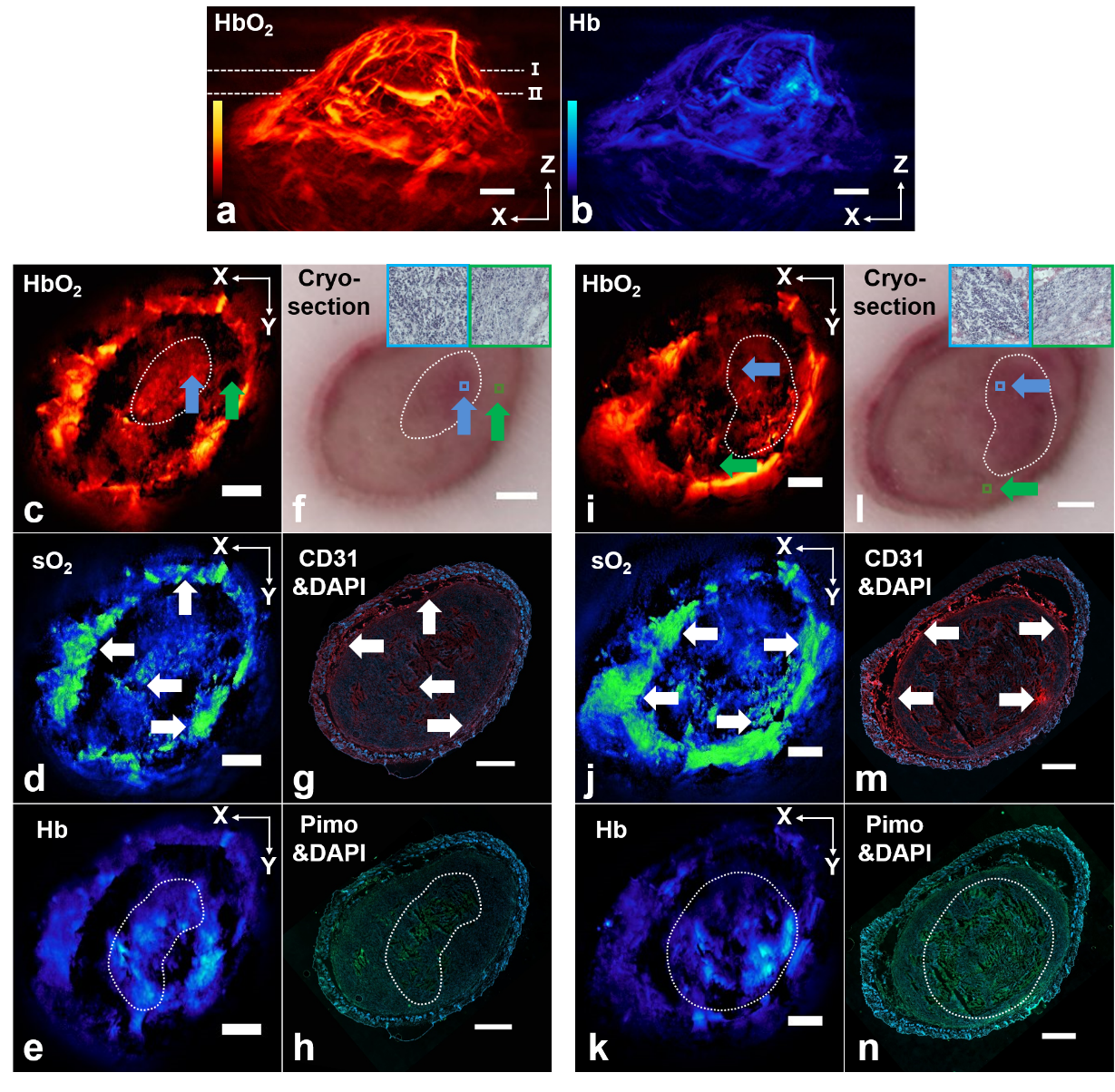


**Fig. S5. Validation of whole-tumour MSOM measurements against histology and immunochemistry of cryosections (KPL4 tumour).** Results are shown for one KPL4 tumour. **(a-b)** Maximum intensity projections in the *xz* plane for HbO_2_ and Hb. **(c-n)** Cryosections were taken at depths I and II from within the tumour regions used to calculate the maximum intensity projections in the *xy* plane for HbO_2_, sO_2_ and Hb, and the sections were stained with hematoxylin-eosin, antibody against endothelial marker CD31 or antibody against Pimonidazole (Pimo)-protein adducts, which mark areas of hypoxia. In some cases, the nuclear stain DAPI (blue) was also included. White arrows and dashed lines highlight features or regions correlating well between optoacoustic images and fluorescence or histology micrographs. Green and blue arrows show the regions with opposite features in HbO_2_ and cryosection images. Higher-magnification views of the cryosection regions boxed in green or blue have been shown, after staining with hematoxylin-eosin. Some images have been rotated in order to highlight correlations with other images. All maximum intensity projections in this figure were calculated over sections with a thickness of 400 μm. Scale bars, 1 mm.

**
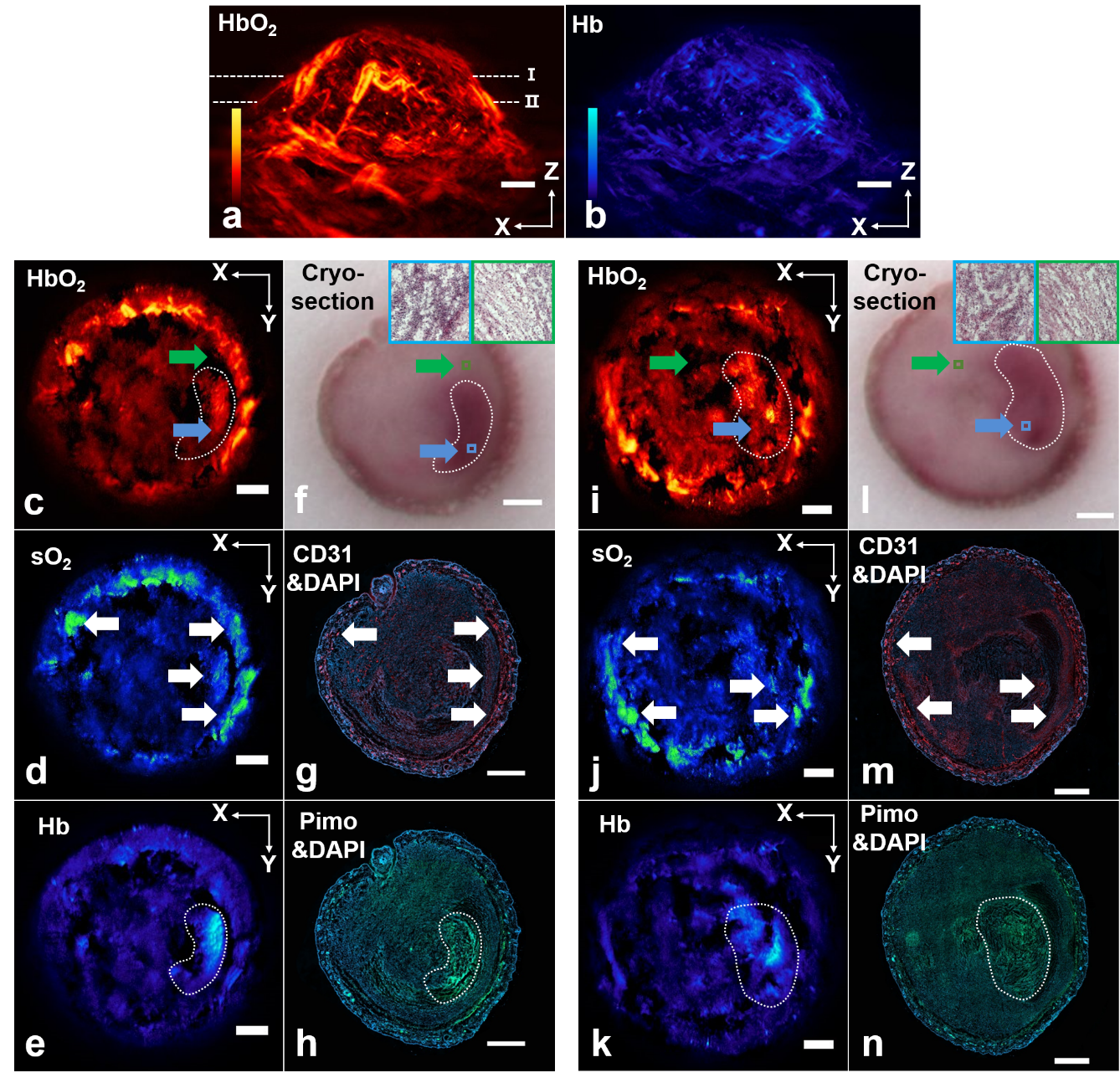
**

**Fig. S6. Validation of whole-tumour MSOM measurements against histology and immunochemistry of cryosections (MDA-MB-231 tumour).** Results are shown for one MDA-MB-231 tumour. **(a-b)** Maximum intensity projections in the *xz* plane for HbO_2_ and Hb. **(c-n)** Cryosections were taken at depths I and II from within the tumour regions used to calculate the maximum intensity projections in the *xy* plane for HbO_2_, sO_2_ and Hb, and the sections were stained with hematoxylin-eosin, antibody against endothelial marker CD31 or antibody against Pimonidazole (Pimo)-protein adducts, which mark areas of hypoxia. In some cases, the nuclear stain DAPI (blue) was also included. White arrows and dashed lines highlight features or regions correlating well between optoacoustic images and fluorescence or histology micrographs. Green and blue arrows show the regions with opposite features in HbO_2_ and cryosection images. Higher-magnification views of the cryosection regions boxed in green or blue have been shown, after staining with hematoxylin-eosin. Some images have been rotated in order to highlight correlations with other images. All maximum intensity projections in this figure were calculated over sections with a thickness of 400 μm. Scale bars, 1 mm.


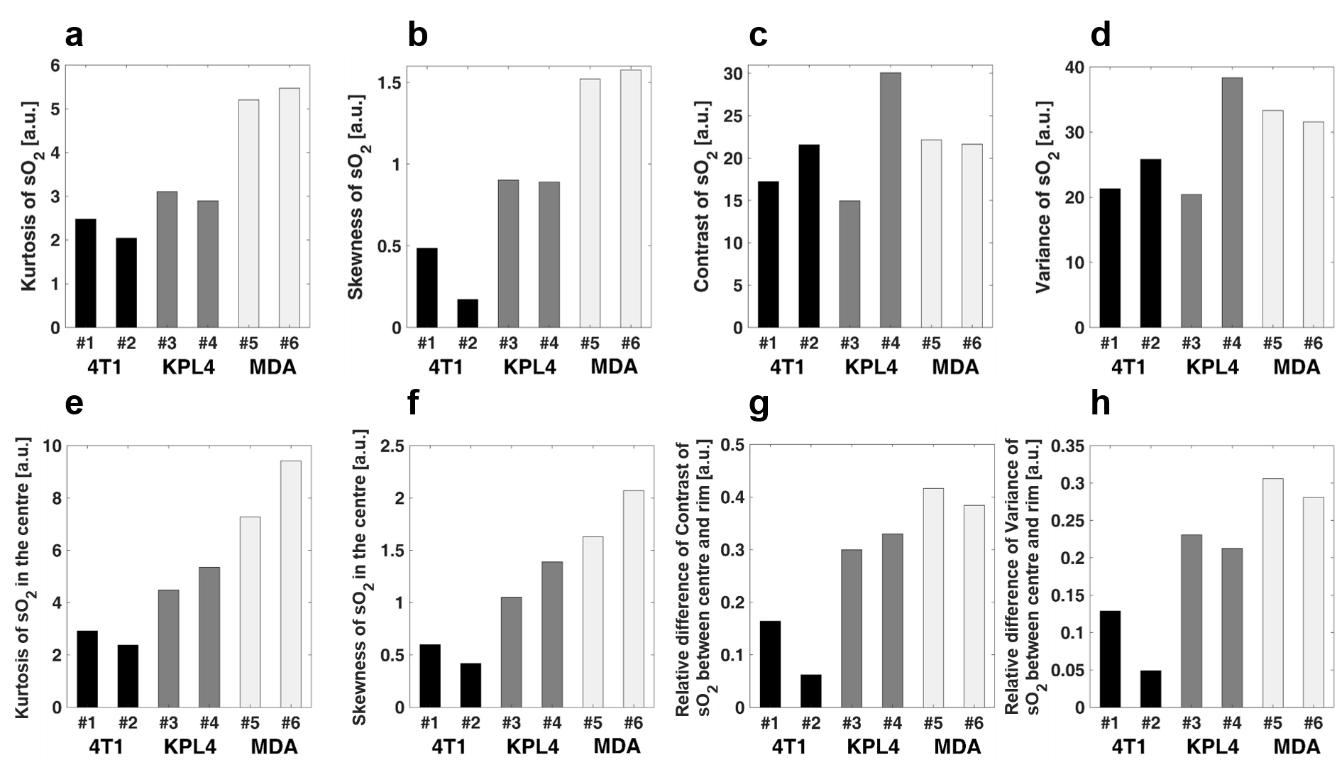


**Fig. S7. MSOM-based *in vivo* quantitation of sO_2_ heterogeneity in entire tumours**. Various heterogeneity metrics were measured in two biological replicates of three types of breast cancer tumours. Details of how metrics were calculated can be found in Methods, together with an explanation of how tumours were segmented into centre and rim regions. MDA refers to MDA-MB-231.

**
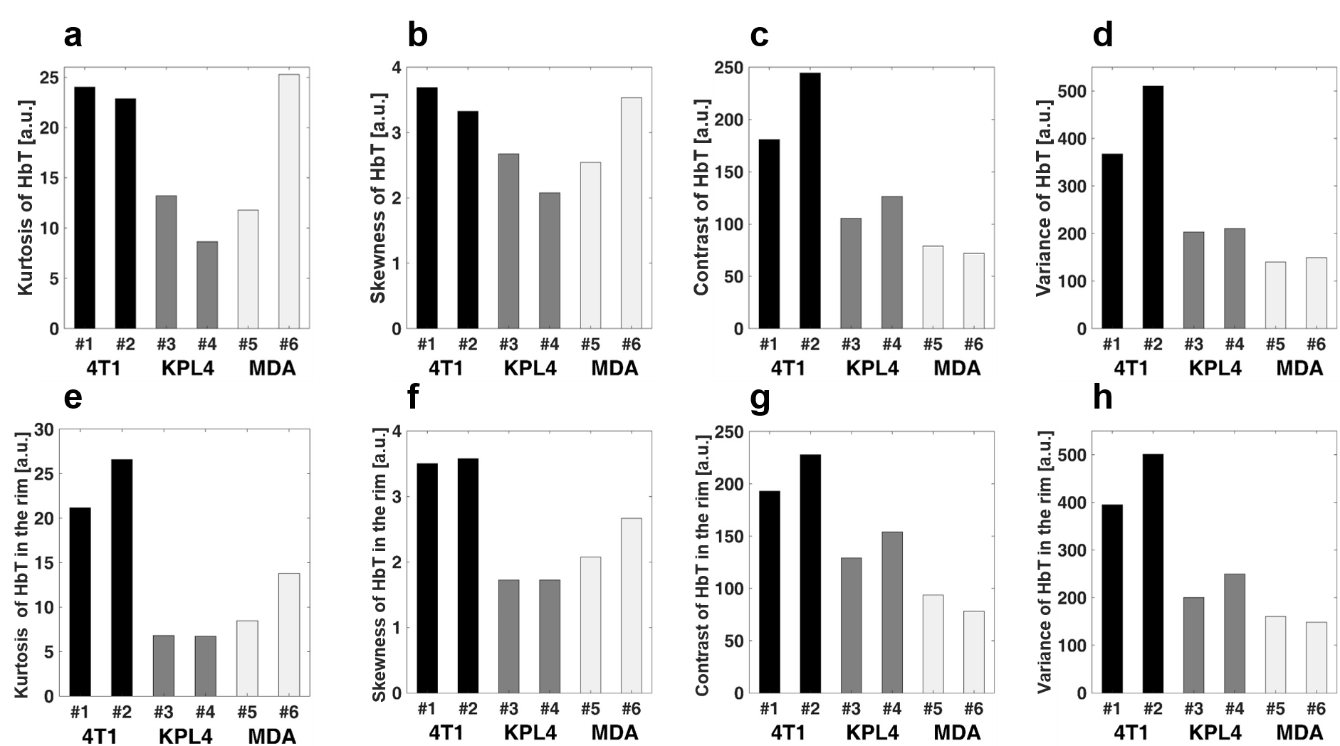
**

**Fig. S8. MSOM-based *in vivo* quantitation of HbT heterogeneity in entire tumours**. Various heterogeneity metrics were measured in two biological replicates of three types of breast cancer tumours. Details of how metrics were calculated can be found in Methods, together with an explanation of how tumours were segmented into centre, middle and rim regions. MDA refers to MDA-MB-231.

**
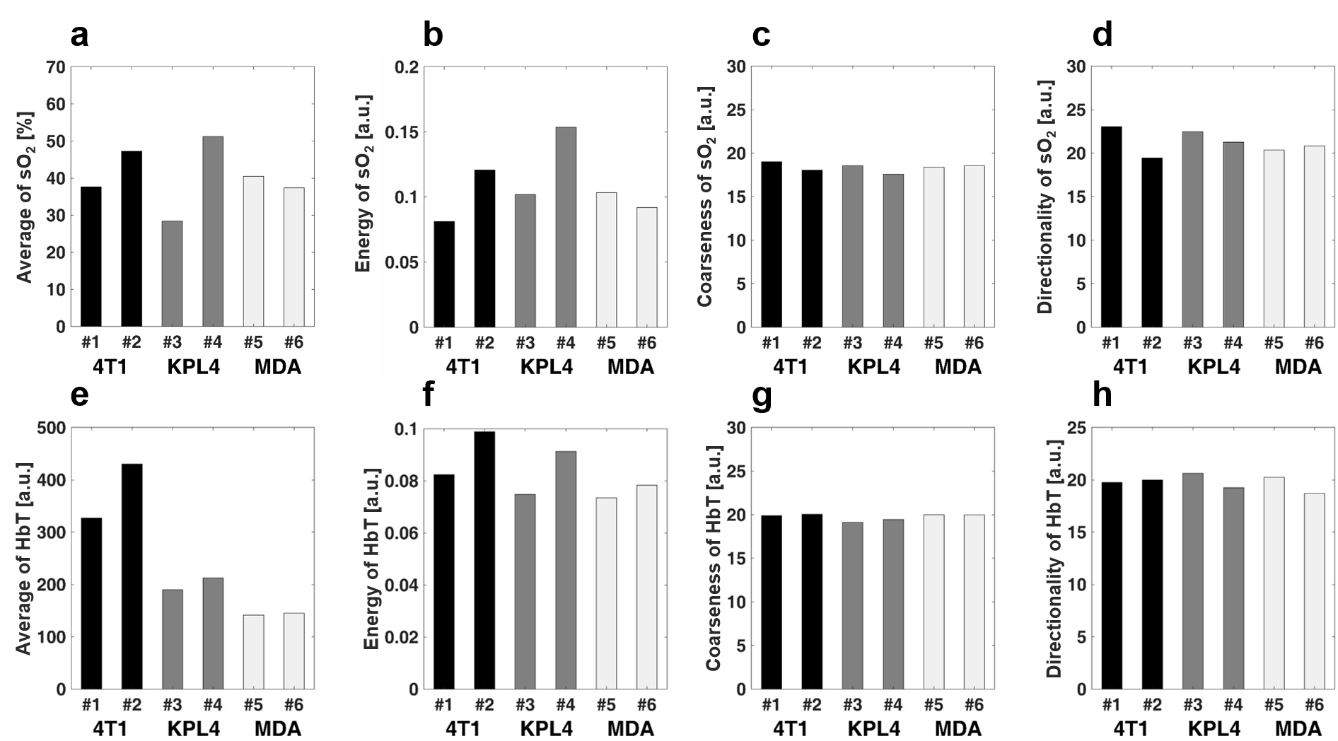
**

**Fig. S9. MSOM-based *in vivo* quantitative metrics in entire tumours**. Various metrics were measured in two biological replicates of three types of breast cancer tumours. Details of how metrics were calculated can be found in Methods. MDA refers to MDA-MB-231.

**
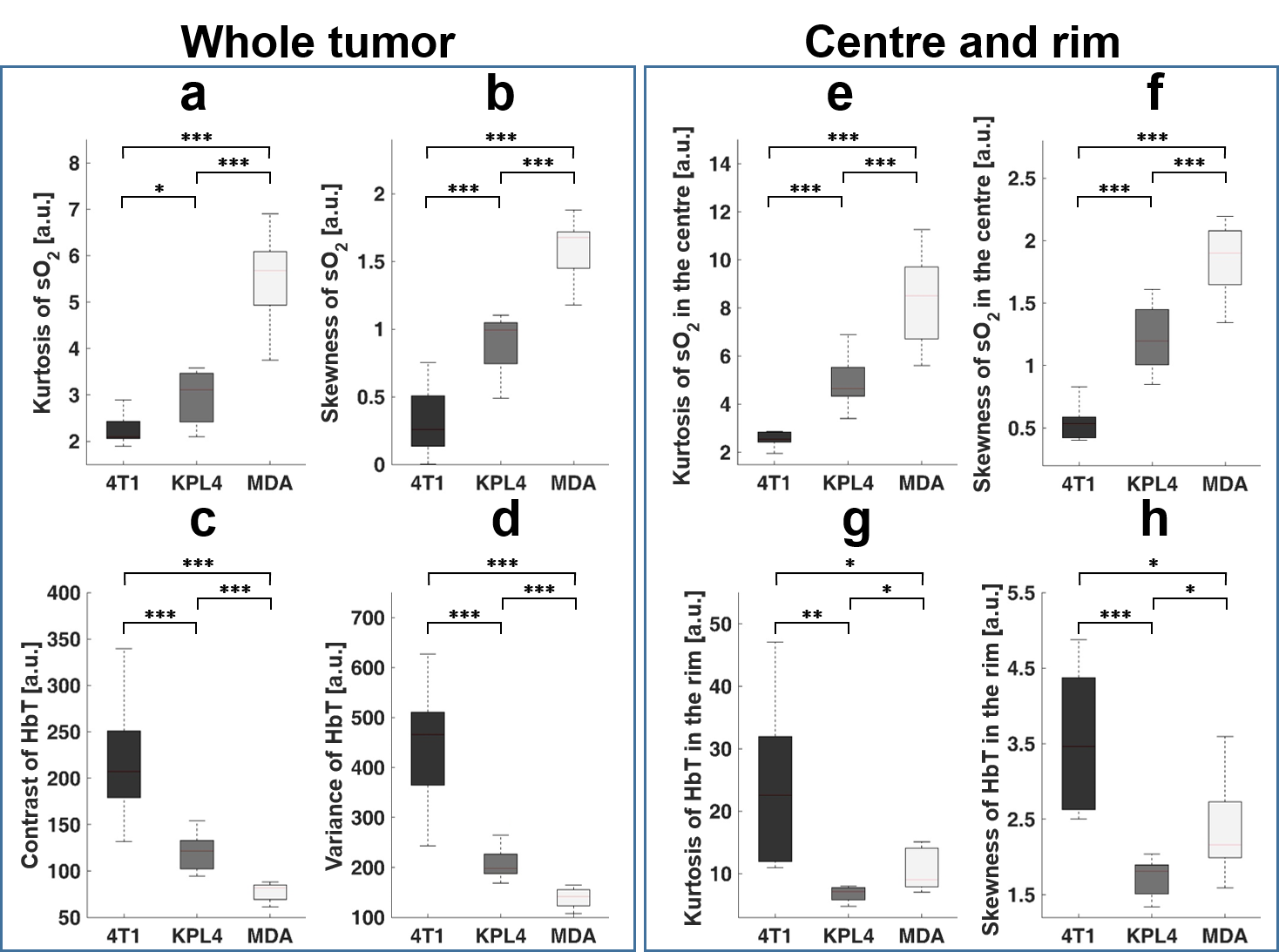
**

**Fig. S10. Comparison of MSOM-based *in vivo* quantitation of sO_2_ and HbT heterogeneity in xy plane sections at different tumour depths through the entire tumours. (a-h)** Various heterogeneity metrics were measured in three types of breast cancer tumours. Details of how metrics were calculated can be found in Methods, together with an explanation of how tumours were segmented into centre and rim regions. MDA refers to MDA-MB-231. * p<0.05, ** p<0.01, *** p<0.001 by unpaired two-tailed t-test (unequal variances). Box between 25^th^ and 75^th^ percentile, line at median.

**
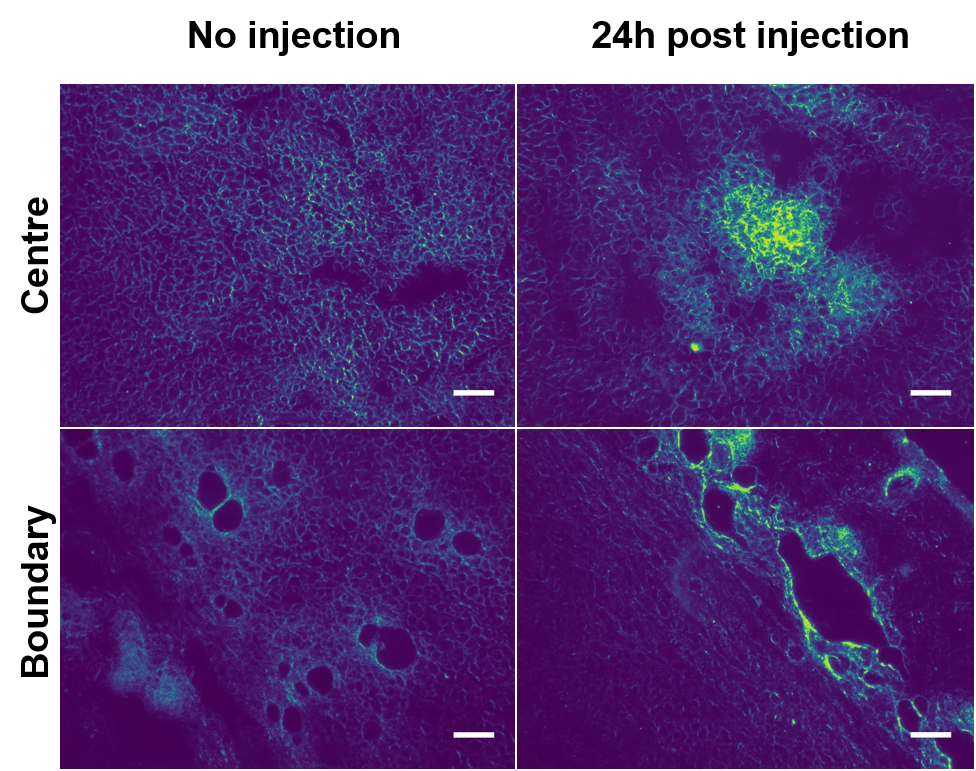
**

**Fig. S11. Validation of the gold nanoparticle experiment based on dark-field microscopy.** Cryosections of 4T1 tumours were imaged after removal from mice that were or were not injected with gold nanoparticles and then sacrificed 24 h later. Nanoparticles appear yellow in the images, while tumour cells appear green. Images were taken from the central region and periphery of the tumour. Magnification, 20X. Scale bar, 50 μm.


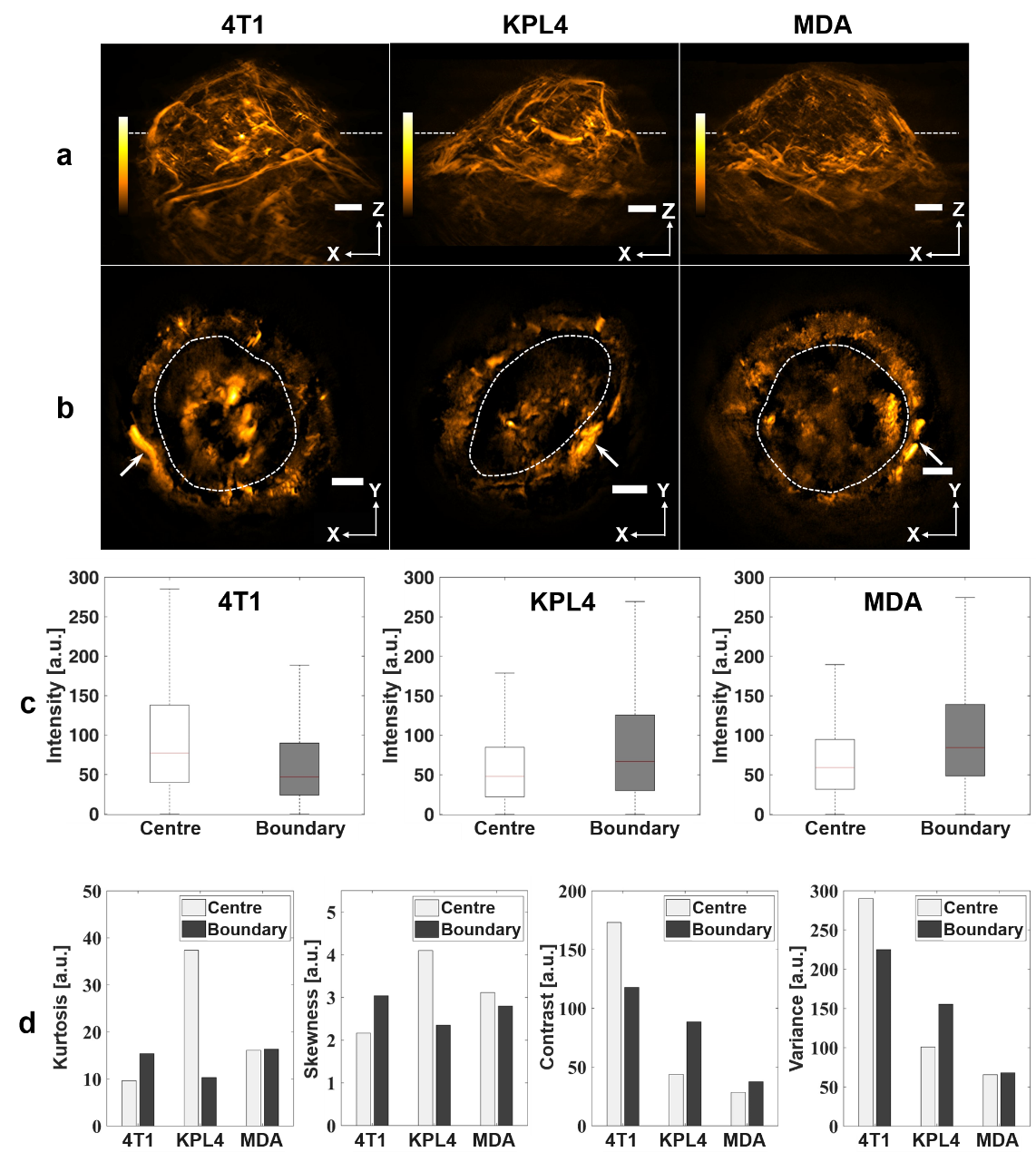


**Fig. S12. Comparison of *in vivo* MSOM imaging of nanoparticle perfusion in three breast cancer tumour types.** **(a)** Maximum intensity projections of gold nanoparticles (AuNPs) in the *xz* plane of entire tumours *in vivo* at 24 h after injection. **(b)** Maximum intensity projections in the *xy* plane at tumour depths defined by the dashed lines in panel (a). Maximum intensity projections were calculated over sections 400 μm thick. **(c)** Quantitation of AuNP intensity and **(d)** various heterogeneity metrics in the centre and boundary of the tumours at depths shown in panels (b). Dashed circles define the edge between tumour centre and boundary. Scale bars, 1 mm.

**References**

1 Gonzalez, R. C. & Woods, R. E. Digital Image Processing. 3rd edn. (Upper Saddle River: Prentice Hall, 2008), 828-836.

2 Tamura, H., Mori, S. & Yamawaki, T. Textural features corresponding to visual perception. *IEEE Transactions on Systems*, *Man*, *and Cybernetics* **8**, 460-473 (1978).

3 Tzoumas, S. *et al*. Unmixing molecular agents from absorbing tissue in multispectral optoacoustic tomography. *IEEE Transactions on Medical Imaging* **33**, 48-60 (2014).
